# Less Radical Surgery for Patient With Early-Stage Cervical Cancer

**DOI:** 10.5812/ircmj.5057

**Published:** 2013-07-05

**Authors:** Zohreh Yousefi, Zahra Kazemianfar, Sima Kadghodayan, Malieheh Hasanzade, Mahmoudreza Kalantari, Mansoureh Mottaghi

**Affiliations:** 1 Department of Obstetrics and Gynecology, Ghaem Hospital, Mashhad University of Medical Sciences, Mashhad, IR Iran; 2 Department of Pathology, Ghaem Hospital, Mashhad University of Medical Sciences, Mashhad, IR Iran

**Keywords:** Cervical Carcinoma, Hysterectomy, Pregnancy, Fertility

## Abstract

**Introduction:**

Surgery in cervical cancer should be used with intention of cure. Radical abdominal trachelectomy is a feasible operation for selected patients with stage Iα-1β cervical cancer which fertility can be preserved.

**Case Report:**

A 30-years-old woman with squamous cell cervical cancer stage (1 A II) diagnosed at September 2011 expressed a wish for fertility-sparing treatment. Radical abdominal hysterectomy and pelvic and para-aortic lymphadenectomy were performed which showed no evidence of lymphatic metastasis. Subsequently, at last follow-up (5 months post-surgery), good oncologic outcomes were found after this procedure. This was the first case of fertility-sparing radical trachelectomy procedures performed at our institution.

**Conclusions:**

Trachelectomy represents a valuable conservative surgical approach for early stage invasive cervical cancer.

## 1. Introduction

Abdominal radical hysterectomy (RAH) with pelvic lymphadenectomy is the standard treatment for early stage (Up to- 1IB) cervical cancer. Management of women with non-metastatic invasive cervical cancer depends to the FIGO stage of the disease at diagnosis. Optimal treatment modality for the women with early-stage cervical cancer (stages IA1-IB1) who have strong desire to maintain fertility is radical trachelectomy (RT) (1).

This procedure is performed with division of the uterus underneath the isthmus, and at the completion of the procedure, the uterus is sutured to the vagina. The technique is satisfying as a wide margin around the lesion is obtained containing the parametria and the upper vagina, but leaving the body of the uterus in situ. Intraoperative mandatory frozen section analysis should be performed on both nodal tissue and upper endo-cervical margins of the trachelectomy specimen (2). Eligibility criteria for this procedure is desire to preserve fertility, up to FIGO stages IB1, limited endo-cervical involvement, no evidence of pelvic lymph node metastasis, and patients < 40 years with tumor size < 2cm) (3). Radical trachelectomy can be performed either vaginal or abdominal. Potential benefits of the abdominal approach for radical trachetectomy includes wider parametrial resection, possible lower intraoperative complication rates, and techniques familiar to most gynecologic oncologists (4). First announcement of radical vaginal trachelectomy (RVT) was reported in 1994 by Dargent; he concluded that it appears that with RVT's overall recurrence and death rates were similar to early-stage cervical cancer treated by radical hysterectomy (RH) or radiotherapy. Furthermore, fertility results of RVT seem to be promising (5). In a meta-analysis of 587 participants to assess the efficacy and safety of radical trachelectomy (RT) and radical hysterectomy (RH) for the patients with early cervical cancer showed that there was no significant difference between two groups in recurrence rate, five-years-free survival rate, and overall survival rate. However, RT compared with RH reduced blood loss and postoperative mortality, and intraoperative complications (6). Moreover, RT may achieve to normal conception rates, while RH makes patients sterile (7). Diaz et al. (8), reported oncologic outcome of fertility-sparing by radical trachelectomy versus radical hysterectomy for stage IB1 cervical carcinoma. For the patients with radical trachelectomy appears to provide equivalent oncological safety to a standard radical hysterectomy. LVSI and DSI appear to be more valuable predictors of outcome than tumor diameter in this subgroup of patients. Concerns about fertility preservation in young women with cervical cancer are particularly common in our country. We report the first case of early-stage cervical cancer as FIGO Stage I a2 who underwent radical abdominal trachelectomy procedures in our institute.

## 2. Case Presentation

**Figure 1.**
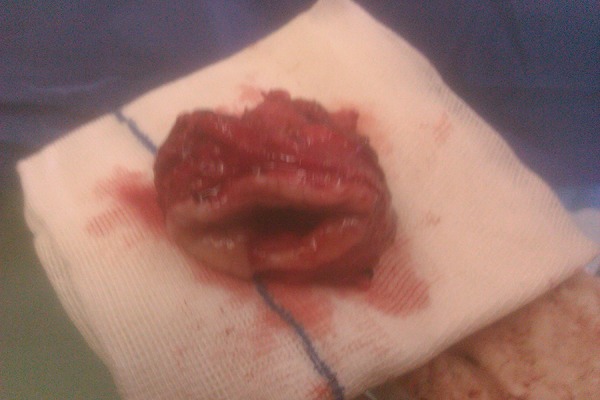
Macroscopic Picture of Trachelectomy Specimen

**Figure 2.**
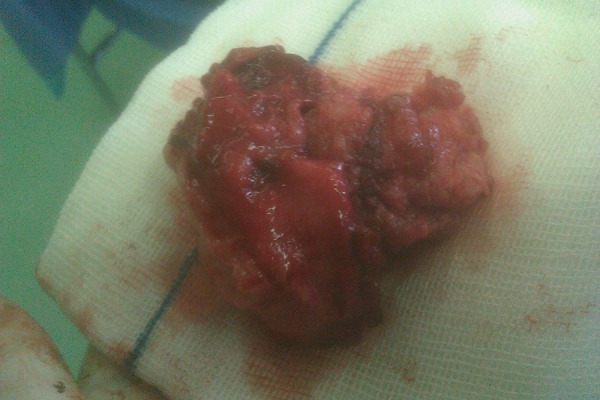
Macroscopic Picture of Trachelectomy Specimen

A 30-years-old woman gravid-2, Para-2, with history of abnormal smear (HSIL) referred to the division of tumor clinic of gynecologic oncology of Ghaem Hospital, Mashhad University of Medical Sciences in September 2011. She was noted that have a cervix with normal appearance. Evidence of colposcopical evaluation showed abnormal vessel and biopsy under colposcopy reported squamous cell cervical cancer as FIGO Stage I a2 at time of diagnosis. She wanted fertility-sparing treatment despite two children. In CT-scan assessment, we had not observed any evidence of lymphatic metastasis in pelvic and para-aortic area. So, she underwent radical abdominal hysterectomy and systematic bilateral pelvic and para-aortic lymphadenectomy. After detecting of negative LNs, the surgical resection of the affected cervix, parametrium and the 1/3 upper vagina with an adequate margin, and 2/3 of the cardinal and uterosacral ligaments were performed. The operation was followed by an endocervical and endometrial sampling above the radical trachelectomy level with evaluation of frozen section pathologic specimen. Finally, after isthmic cerclage-vaginal anastomos to Isthmus was carried out (Figures 1 and 2). At last follow-up (5 months post-surgery), she had good oncologic outcomes and no evidence of recurrence of disease was found. 

## 3. Discussion

Radical trachelectomy is a valuable option for women with early-stage of cervical cancer. Previous studies reported that the recurrence rates of cervical cancer in patients who underwent RAT were comparable to patients who carried out RAH. These recurrence rates ranged from 0% to 8% (9). Kioliopoulos et al. (10), reported that 210 women who underwent radical trachelectomy had 35 live births after surgery. Radical trachelectomy can be performed even with preservation of the concurrent pregnancy and associated with successful pregnancy outcome (11). The psychosocial impact of cancer-related infertility is important because a high proportion of these patients experience feelings of depression. Preoperative consultation or immediate postoperative support about assessing the emotional, sexual, and quality of life of women undergoing radical trachelectomy versus radical hysterectomy for treatment of early-stage cervical cancer improved the patient’s life especially during the first year (12). A recent review literature has reported 70% pregnancy rate in the women who wanted to conceive following RVT (13). We must notice that such patients should be informed about the risk of second trimester loss and preterm delivery (14). Problems associated with RT included menstrual/vaginal issues, emotional impact, and life interruptions/return to normalcy, general pain, and recovery process. The PCL identified higher rates of neo-cervical stenosis (58%), encroachment (54%), vaginal scarring (50%), and dyspareunia (33%), and increased documentation of reproductive consults (54%) (15). After surgery, contraception is recommended for 6-12 months. Close surveillance should be instituted with scheduled papanicoloau testing, colposcopic evaluation, and endocervical curettage carried out every 3 months in first year, 4 months in second year, 6 months from 2-5 years and annually thereafter till 10 years (12). Identification of optional treatment requires further reports with larger sample sizes and longer follow-up periods. More conservative methods have emerged as alternative treatment modalities for younger women who are desirable for the preservation of fertility without having a considerable adverse effect on cure rates. Radical trachelectomy is currently the most available method.

## References

[1] Danska-Bidzinska A, Sobiczewski P, Bidzinski M, Gujski M (2011). [Radical trachelectomy--retrospective analysis of our own case material].. Ginekol Pol..

[2] Chen Y, Xu H, Zhang Q, Li Y, Wang D, Liang Z (2008). A fertility-preserving option in early cervical carcinoma: laparoscopy-assisted vaginal radical trachelectomy and pelvic lymphadenectomy.. Eur J Obstet Gynecol Reprod Biol..

[3] Lanowska M, Morawietz L, Sikora A, Raber G, Mangler M, Speiser D (2011). Prevalence of lymph nodes in the parametrium of radical vaginal trachelectomy (RVT) specimen.. Gynecol Oncol..

[4] Gottschalk E, Mangler M, Schneider A, Koehler C, Lanowska M (2011). Pregnancy after lymphadenectomy and neoadjuvant chemotherapy followed by radical vaginal trachelectomy in FIGO stage IB1 cervical cancer.. Fertil Steril..

[5] Dursun P, LeBlanc E, Nogueira MC (2007). Radical vaginal trachelectomy (Dargent's operation): a critical review of the literature.. Eur J Surg Oncol..

[6] Xu L, Sun FQ, Wang ZH (2011). Radical trachelectomy versus radical hysterectomy for the treatment of early cervical cancer: a systematic review.. Acta Obstet Gynecol Scand..

[7] Cibula D, Pinkavova I, Dusek L, Slama J, Zikan M, Fischerova D (2011). Local control after tailored surgical treatment of early cervical cancer.. Int J Gynecol Cancer..

[8] Diaz JP, Sonoda Y, Leitao MM, Zivanovic O, Brown CL, Chi DS (2008). Oncologic outcome of fertility-sparing radical trachelectomy versus radical hysterectomy for stage IB1 cervical carcinoma.. Gynecol Oncol..

[9] Du XL, Sheng XG, Jiang T, Li QS, Yu H, Pan CX (2011). Sentinel lymph node biopsy as guidance for radical trachelectomy in young patients with early stage cervical cancer.. BMC Cancer..

[10] Koliopoulos G, Sotiriadis A, Kyrgiou M, Martin-Hirsch P, Makrydimas G, Paraskevaidis E (2004). Conservative surgical methods for FIGO stage IA2 squamous cervical carcinoma and their role in preserving women's fertility.. Gynecol Oncol..

[11] Sioutas A, Schedvins K, Larson B, Gemzell-Danielsson K (2011). Three cases of vaginal radical trachelectomy during pregnancy.. Gynecol Oncol..

[12] Abu-Rustum NR, Neubauer N, Sonoda Y, Park KJ, Gemignani M, Alektiar KM (2008). Surgical and pathologic outcomes of fertility-sparing radical abdominal trachelectomy for FIGO stage IB1 cervical cancer.. Gynecol Oncol..

[13] Carter J, Sonoda Y, Chi DS, Raviv L, Abu-Rustum NR (2008). Radical trachelectomy for cervical cancer: postoperative physical and emotional adjustment concerns.. Gynecol Oncol..

[14] Kim JH, Park JY, Kim DY, Kim YM, Kim YT, Nam JH (2010). Fertility-sparing laparoscopic radical trachelectomy for young women with early stage cervical cancer.. BJOG..

[15] Carter J, Raviv L, Sonoda Y, Chi DS, Abu-Rustum NR (2011). Recovery issues of fertility-preserving surgery in patients with early-stage cervical cancer and a model for survivorship: the physician checklist.. Int J Gynecol Cancer..

